# Microbiota disbiosis is associated with colorectal cancer

**DOI:** 10.3389/fmicb.2015.00020

**Published:** 2015-02-02

**Authors:** Zhiguang Gao, Bomin Guo, Renyuan Gao, Qingchao Zhu, Huanlong Qin

**Affiliations:** Department of General Surgery, Shanghai Jiao Tong University Affiliated Sixth People's HospitalShanghai, China

**Keywords:** colorectal cancer, proximal colon, distal colon, mucosa-associated microbiota, gut dysbiosis

## Abstract

The dysbiosis of the human intestinal microbiota is linked to sporadic colorectal carcinoma (CRC). The present study was designed to investigate the gut microbiota distribution features in CRC patients. We performed pyrosequencing based analysis of the 16S rRNA gene V3 region to investigate microbiota of the cancerous tissue and adjacent non-cancerous normal tissue in proximal and distal CRC samples. The results revealed that the microbial structures of the CRC patients and healthy individuals differed significantly. Firmicutes and Fusobacteria were over-represented whereas Proteobacteria was under-represented in CRC patients. In addition, *Lactococcus* and *Fusobacterium* exhibited a relatively higher abundance while *Pseudomonas* and *Escherichia-Shigella* was reduced in cancerous tissues compared to adjacent non-cancerous tissues. Meanwhile, the overall microbial structures of proximal and distal colon cancerous tissues were similar; but certain potential pro-oncogenic pathogens were different. These results suggested that the mucosa-associated microbiota is dynamically associated with CRC, which may provide evidences for microbiota-associated diagnostic, prognostic, preventive, and therapeutic strategies for CRC.

## Introduction

Colorectal cancer is the third most common cancer and the fourth leading cause of cancer deaths worldwide, accounting for approximately 1.2 million new cases and 600,000 deaths per year (Brenner et al., 2014). Based on embryological, morphologic, physiological, and biochemical differences in anatomic sites, Bufill proposed the existence of distinct categories of CRC according to the location of the tumor in the proximal (right) or distal (left) segments relative to the splenic flexure. Epidemiologic studies showed the difference in terms of incidence of CRC (Bufill, 1990). For example, rectal cancer accounted for beyond 50% in proportion of all CRC in some south, central Asia and South America countries, whereas the incidence of colon cancer is beyond of rectum cancer in the countries of Europe, North America (Takada et al., 2002). Many risk factors are associated with CRC, including inflammatory bowel disease, smoking, excessive alcohol consumption, high consumption of red and processed meat, obesity, and diabetes. For example, high dietary fat intake has been reported to increase the risk of proximal cancers while high protein intake increases the incidence of distal cancers (McMichael and Potter, 1985; West et al., 1989). Another case-control study of Chinese who reside in North America show high carbohydrate intake is associated with increased right colon cancer in women but increased rectal cancer amongst men (Borugian et al., 2002). At present, there is substantial evidence suggesting that the environmental factors mentioned above markedly affect the intestinal microbiota composition (Wu et al., 2011). Moreover, there is unequivocal evidence linking gut dysbiosis to CRC development (Schwabe and Jobin, 2013). It is evaluated that the human gastrointestinal tract harbors approximately 1000 species of bacteria estimating 10^14^ cells, which constitute about 90% of all cells in the human body (Qin et al., 2010). In addition to influencing host nutrition via metabolism, the intestinal microbiota can play both beneficial and detrimental roles by controlling epithelial proliferation and differentiation (Srikanth and McCormick, 2008).

Accumulated studies show that several bacterial species seem to involve in pathogenesis of CRC (Srikanth and McCormick, 2008; Castellarin et al., 2011; Kostic et al., 2011; Marchesi et al., 2011). *Streptococcus Gallolyticus* (Formerly *Streptococcus bovis*) is present approximately 20–50% of colon tumors and less than 5% in the normal colon. Wei and his colleagues report that the abundance of *Ruminococcus obeum* and Allobaculum-like bacteria are increased in the feces of 1,2-dimethyl hydrazine (DMH)-treated rats developing precancerous mucosal lesions (Wei et al., 2010). In addition, a significant elevation of the *Bacteroides*/*Prevotella* population is reported. *Bifidobacterium longum*, *Clostridium clostridioforme*, and *Ruminococcus bromii* are under-represented in RC patients compared to healthy individuals (Sobhani et al., 2011). *Fusobacterium nucleatum* is found over-represented in tumor micro- environment (Ray, 2011). Recent studies have provided mechanistic evidence for the involvement of gut bacteria in the development of CRC. Animal experiment reveals that mutant mice that are genetically susceptible to CRC develop significantly fewer tumors under germ-free conditions than when they have a conventional microbiota (Uronis et al., 2009). Extracellular genotoxins and DNA damaging superoxide radicals produced by *Enterococcus faecalis* can contribute to CRC development (Huycke et al., 2002; Wang and Huycke, 2007; Wang et al., 2008). DNA damage also can be induced by genotoxic *Escherichia coli* which harbor the polyketide synthetase (pks) island and encode a genotoxin called colibactin (Nougayrède et al., 2006;Cuevas-Ramos et al., 2010).

However, previous studies have suggested that different bacterial species preferentially inhabit the tumor sites. It is not yet clear whether the over-representation or under-representation of particular microbial species in tumor microenvironment is indicative of a contributory role in the development of CRC. Although a causal role of intestinal microflora in CRC development has not been demonstrated, evidence based on bacterial culture indicated that some potential pro-oncogenic pathogens, which may be the members of commensals, contribute to tumor initiation and development.

In this study, we performed pyrosequencing based analysis of 16S rRNA genes to analyze the overall structure of microbiota in patients with CRC and in healthy controls. We first found that a significant difference in intestinal bacterial flora was existed between the healthy individuals and CRC patients. We further demonstrated that the composition of the tumor microbiome differed from that of adjacent non-neoplastic tissue. We also determined the subsite-specific alterations in the CRC microbiota. The results of these studies provide evidence supporting that these bacteria could be used for microbiota-associated diagnosis, prognosis prevention and treatment for CRC.

## Materials and methods

### Ethics statement

All study protocols were reviewed and approved by the Ecthics Committee of Shanghai Jiaotong University Affiliated Sixth People's Hospital and informed consent was provided by each patient following the protocol approved by the Institutional Review Board.

### Sample collection and DNA extraction

Colorectal cancerous mucosa tissue samples were obtained intraoperatively from recently diagnosed CRC patients [31 cancerous tissues (T), 20 adjacent non-cancerous tissues (5 cm from the cancerous tissue; P), 15 proximal colon cancer tissues (T_p_), 16 distal colon cancer tissues (T_d_)]. Proximal colon cancers were located in the ascending colon (5–10 cm from ileal valve); distal colon cancers were located in the sigmoid colon (25–35 cm from anus). Additionally, 30 corresponding colorectal mucosal samples of healthy volunteers [15 proximal colon tissues (H_p_) and 15 distal colon tissues (H_d_)] were collected during colonoscopy (Table 1). All participants who met any of the exclusion criteria as described were not enrolled in this study (Table 2), including use of antibiotics within 2 months, and regular use of Non-steroidal antiinflammatory drugs **(NSAIDS)**, statins, or probiotics. Individuals that complicated with actue/chronic intestinal obstruction, chronic bowel disorders, and other foci of infections or food allergies/dietary restrictions were also excluded from the study. Additional exclusion for CRC patients included chemotherapy or radiation treatments prior to surgery. All participants received conventional bowel preparation without preoperative antibiotics administration. Samples were transported to the laboratory within 30 min after collection by study participants. DNA was extracted from all samples using MoBio Powersoil DNA extraction kits (MoBio, Carlsbad, CA) according to the manufacturer's instructions and stored at −20°C prior to amplification steps.

**Table 1:** **Summary information of individuals in the study**.

**Group**	**H**	**T**	
**Sample**	**Tissue**	**Tissue**	***P*-value**
No.	30	31	>0.05
Male/female	14/16	15/16	>0.05
Age (year)	70 ± 5.1	67 ± 7.2	>0.05
BMI(kg/m2)	22.2 ± 2.2	24.5 ± 4.3	>0.05
Stage (A/B/C)^†^		8/15/8	>0.05
**LOCATION**			
Proximal colon	15	14	>0.05
Distal colon	15	17	>0.05
Preoperative albumin (g/dL)	42.2 ± 2.6	36.5 ± 3.4	>0.05
Preoperative Hb (g/L)	126 ± 12.4	123.2 ± 19.6	>0.05
Creatinine (mg/dL)	1.1 ± 0.2	1.2 ± 0.13	>0.05

*Abbreviations: BMI, body mass index,; No, number of participants*.

† *Dukes staging*.

**Table 2:** **Inclusion and exclusion criteria of the individuals in the study**.

**CRC patients**	**Healthy individuals**
**INCLUSION CRITERIA**	**INCLUSION CRITERIA**
Age 40–75 years	Age 40–75 years
Diagnosis was confirmed by biopsy and histological analysis	BMI 18.5–30 kg/m^2^
Undergone radical resection and no distant metastasis (including liver)	
**EXCLUSION CRITERIA**	**EXCLUSION CRITERIA**
Age >75 years	BMI >30 kg/m^2^
Pregnancy	Pregnancy
Known lactose intolerance	Known lactose intolerance
Clinically significant immunodeficiency	Clinically significant immunodeficiency
Usage of antibiotics and additional gastrointestinal disorders (e.g., Crohn's disease or ulcerative colitis)	Usage of antibiotics and additional gastrointestinal disorders (e.g., Crohn's disease or ulcerative colitis)
Received antibiotics for the past 3 months before surgery	Received antibiotics for the past 3 months before surgery
Evidence of infection	Evidence of infection
Probiotics or prebiotics and excessive fiber intake within 2 weeks	Probiotics or prebiotics and excessive fiber intake within 2 weeks
Undergoing emergency operation	Undergoing emergency operation
Bowel preparation for colonoscopy within 6 days prior to surgery	Bowel preparation for colonoscopy within 6 days prior to surgery
Undergoing proctectomy with low rectal anastomosis or surgery for polypoid lesion	Undergoing proctectomy with low rectal anastomosis or surgery for polypoid lesion
Laparoscopic surgery	Laparoscopic surgery
Patients received preoperative chemotherapy or radiation therapy	Suffered from other tumor

*Abbreviations: BMI, body mass index; CRC, colorectal cancer*.

### Pyrosequencing analysis

Amplification of the V3 region of the bacterial 16S rRNA gene was performed in triplicate using primers 515F and 806R labeled with 12-bp error correcting Golay barcodes (Kõljalg et al., 2013). Twenty microliter reactions containing 5 Prime Hot Master Mix (5 Prime, Inc., Gaithersburg, MD) were amplified at 94°C for 5 min followed by 35 cycles of 94°C for 1 min, 63°C for 1 min, and 72°C for 1 min followed by a final extension at 72°C for 10 min. Replicate PCR reactions were combined and gel purified using the GenElute Gel Extraction kit (Sigma-Aldrich, St. Louis, MO), followed by an additional purification with AMpure beads (Beckman Coulter, Indianapolis, IN) and quantified with the PicoGreen DNA Assay (Invitrogen, Carlsbad, CA, USA) prior to library pooling. Pyrosequencing was performed by the University of South Carolina's Engencore Sequencing Facility using a 454 Life Sciences GS FLX System with standard chemistry.

### Bioinformatics analysis

In order to gain high-quality and more precise bioinformation, we used effective sequences which contain some point mutation and macromolecular homopolymers (Qiime, version 1.17 http://qiime.org/) (Hamady et al., 2008). The optimized sequences were then clustered into opterational taxonomic units (OTUs) using Usearch (version 7.1 http://qiime.org/) with a criterion of a minimum similarity of 97%. Chimera sequences arising from the PCR amplification were detected and excluded from the OTUs using uchime (version 4.2.40 http://drive5.com/usearch/manual/uchime_algo.html) (Edgar, 2013). Representative OTUs were aligned to the optimized sequences and the abundance of OTUs per samples was obtained for performing the following further analysis.

Applying Bayesian algorithms of RDP classifier to analyse the presentative OTUs at 97% similarity in the following databases: 16S bacteria and archaeal ribosomes Silva (Release115 http://www.arb-silva.de) (Quast et al., 2013); RDP (Release 11.1 http://rdp.cme.msu.edu/) (Cole et al., 2009); Greengene (Release 13.5 http://greengenes.secondgenome.com/) (DeSantis et al., 2006); ITS fungus (Unite Release 5.0 http://unite.ut.ee/index.php); functional genes FGR (Release7.3 http://fungene.cme.msu.edu/) (Fish et al., 2013).

### Statistical analysis

All clinical statistical analyses were performed with the SPSS 18.0 software program for Windows (IBM). Pearson's χ^2^-test and Fisher's exact test were applied to compare qualitative variables, and quantitative variables were analyzed by Student's *t*-test or Spearman's ρ rank correlation coefficient determination. A univariate analysis was performed using the Kaplan–Meier method (the log-rank test). A multivariate analysis was performed using a Cox multivariate proportional hazard regression model in a stepwise manner (backward, conditional). The model included all clinicopathological variables found to have significant prognostic value in the univariate analysis. A two-tailed value of *P* < 0.05 was considered to be statistically significant.

### Data access

The 16S sequence data generated in this study was submitted to the GenBank Sequence Read Archive accession number (SRP037786).

## Results

### Richness and diversity analysis

**Figure 1:**
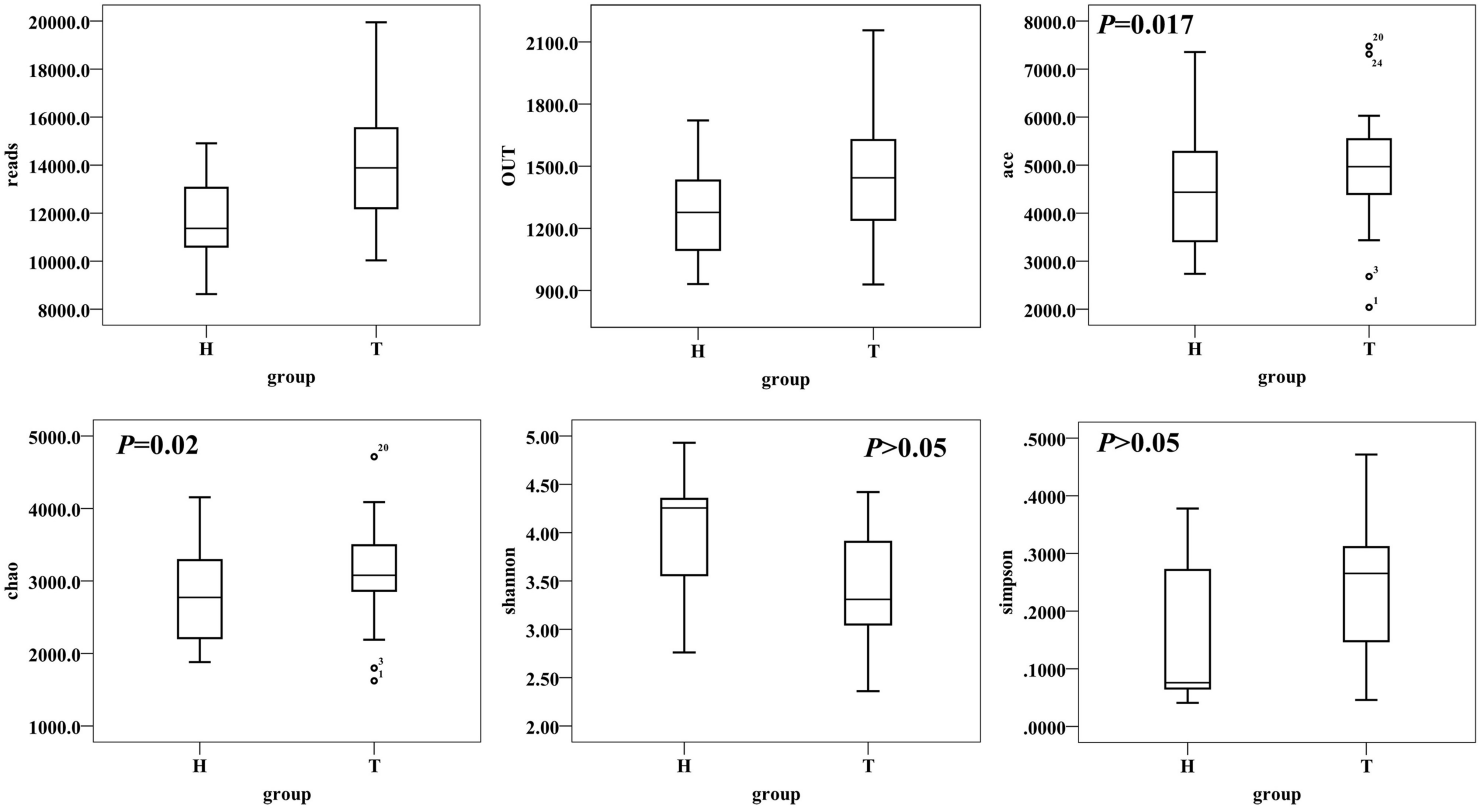
**Alpha-diversity distances calculated using phylotype relative abundance measurements between healthy and CRC groups demonstrate that the microbial richness of CRC patients is higher than healthy individuals, while the diversity has no statistical significance between two groups**.

A total of 1,378,458 high-quality and classifiable reads were obtained from this study, with an average of 17,018 (*n* = 81) reads per sample. At 3% dissimilarity level, a total of 148,959 OTUs in all samples and an average of 1839 OTUs (*n* = 81) per sample were identified. The value of Good's coverage for each group was over 93%, indicating that the 16S rRNA sequences identified in the groups represent the majority of bacteria present in the study samples. Whereas we didn't observe the plateau of the refraction curve (Figure S1) with the current sequencing. We examined the estimators of community richness (Chao and Ace indexes) and diversity and evenness (Shannon and Simpson indexes) in CRC patient and healthy individual samples. While there were statistically significant differences of Shannon and Simpson diversity indexes between CRC and healthy individual [Shannon, 3.43 ± 0.60 vs. 4.01 ± 0.58 (95% CI of the difference, −0.89 to −0.28); *P* < 0.001; Simpson, 0.24 ± 0.12 vs. 0.15 ± 0.12 (95% CI of the difference, 0.04 to 0.16), *P* = 0.002], demonstrating the significantly lower diversity found in CRC than healthy individual, There were no statistically significant differences with Chao and Ace index between the two groups [Chao, 3128 ± 646 vs. 2822 ± 627 (95% CI of the difference, −20.63 to 632.45), *P* = 0.06; Ace, 4900 ± 1129 vs. 4498 ± 1137 (95% CI of the difference, -88.51 to 1072.99), *P* = 0.09, Figure 1].

### Mucosa-associated microbiota in CRC patients and healthy individuals differed significantly

**Figure 2:**
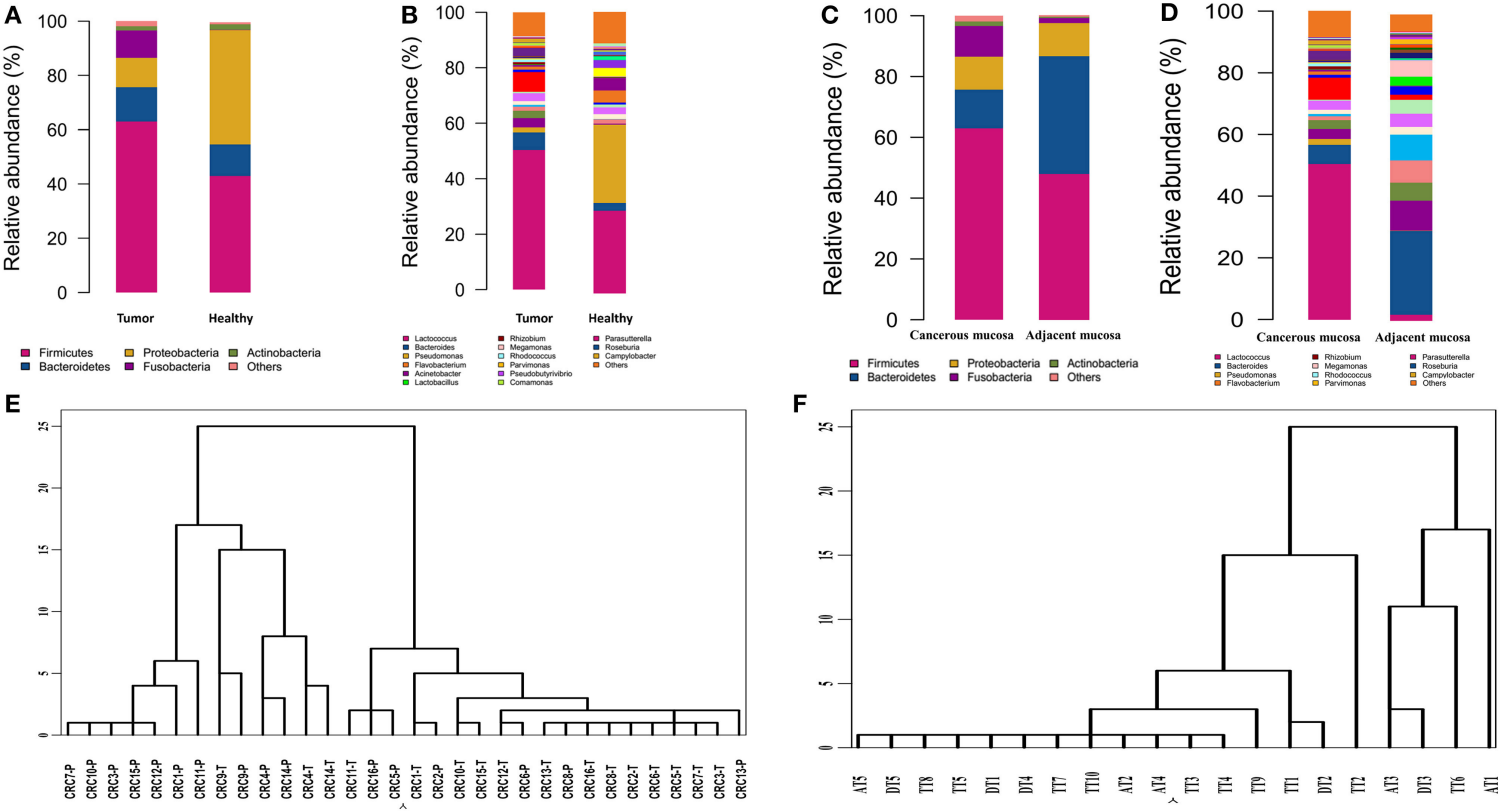
**Different structures of gut microbiota between healthy individuals and CRC patients**. **(A)** The dominant phyla of group tumor and healthy. **(B)** The dominant genera of group tumor and healthy. **(C)** The dominant phyla of group cancer and non-cancerous mucosa. **(D)** The dominant genera of group cancer and cancerous mucosa. **(E)** Hierarchical clustering of phylotype relative abundance measurements demonstrates that microbial composition of tumor samples from different individuals is more highly correlated than tumor/health samples within individuals. **(F)** Hierarchical clustering of proximal and distal CRC.

Bacterial communities from the mucosa of healthy individuals and CRC patients were analyzed. The overall microbial composition for each group at the phylum level is shown in Figure 2A. According to the taxonomic results, we demonstrated that Firmicutes, accounting for 63.46 and 43.36% [95% CI of the difference, 5.67–34.32%] of the gut microbiota in CRC patients and healthy individuals, respectively (*P* < 0.001), was the most predominant phylum in CRC patients. While Proteobacteria were the most predominant phylum in healthy individuals compared to CRC patients with the proportion of 60.35 and 10.66% [95% CI of the difference, −35.32 to −15.57%], respectively (*P* < 0.001). And Bacteroidetes were the secondary phylum in both groups with the proportion of 12.77 and 13.00% [95% CI of the difference, −4.99 to 5.44%], respectively (*P* > 0.05). Finally, Fusobacteria constituted the third most abundant phyla in CRC group, contributing 10.58% compared with 0.03% [95% CI of the difference, 0.25–12.68%] in healthy individuals (*P* < 0.001).

**Figure 3:**
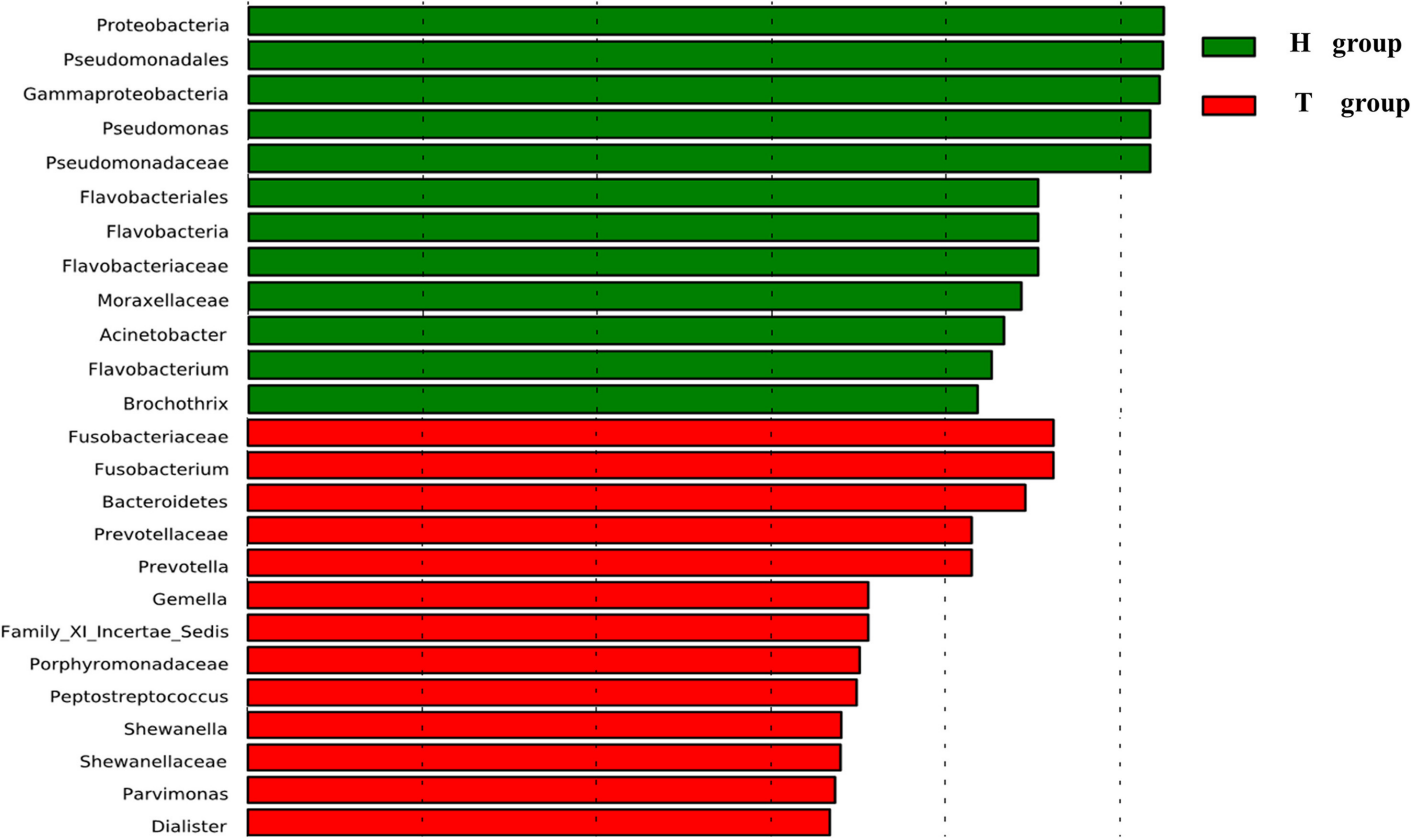
**Histogram of the LDA scores for differentially abundant genera**. Cladogram was calculated by LEfSe, a metagenome analysis approach.

At the genus level, our studies found the microbial composition differed significantly between CRC patients and healthy individuals (Figure 2B). The genera *Lactococcus* [50.85 vs. 25.35% (95% CI of the difference, 8.45–37.26%), *P* = 0.007], *Fusobacterium* [10.08 vs. 0.01% (95% CI of the difference, -0.27 to 10.63%), *P* = 0.032], *Escherichia–Shigella* [2.92 vs. 0.22% (95% CI of the difference, 0.91–5.75%), *P* = 0.004], *Peptostreptococcusten* [0.23 vs. 0.001% (95% CI of the difference, -0.01 to 0.77%), *P* = 0.036] were enriched in CRC patients, however, *Epilithonimonas, Flavobacterium* (*Flavobacteria*)*, Pedobacter, Sphingobacterium* (*Sphingobacteria*), *Caulobacter, Brevundimonas, Sphingomonas, Sphingomonas* (*Alphaproteobacteria*), *Acidovorax, Janthinobacterium* (*Betaproteo- bacteria*), *Buttiauxella, Rahnella, Acinetobacter, Janthinobacterium, Psychrobacter, Pseudomonas, Stenotrophomonas* (*Gammaproteobacteria*), *Psychrobacter, Propioni- bacterium* (*Actinobacteria*) were reduced in CRC patients. Using Linear discriminant analysis (LDA) coupled with effect size measurements (LEfSe), we found that *Fusobacterium, Prevotella* and *Peptostreptococcus* were the key phylotypes that contribute to the dysbiosis of mucosa-associated microbiota in CRC patients, while *Pseudomonas* and *Flavobacterium* were the key phylotypes that contribute to the distribution of mucosa- adherent microbiota in healthy individuals (Figure 3).

### Bacterial community in cancerous tissue and adjacent non-cancerous normal tissue

According to hierarchical clustering analysis, the microbial communities of cancerous tissues are more similar than are non-cancerous tissues and can be distinguished from each other, while the microbial communities of a tumor and matched non-cancerous tissue from a given patient significantly differ from each other (Figure 2E), This finding suggests that there are marked differences in the microbial composition of tumor and non-cancerous tissue.

A taxonomy-based comparison was performed to determine the differences between the microbiota of cancerous tissues and adjacent non-cancerous colorectal tissues. At the phylum level, Firmicutes was the most predominant phylum, contributing 63.46 and 39.54% of the gut microbiota in cancerous tissues and adjacent non-cancerous tissues, respectively, followed by Bacteroidetes, which contributed 12.77 and 19.1%, respectively. Proteobacteria, Fusobacteria, and Actinobacteria constituted the next most dominant phyla, contributing 10.66, 9.58, and 1.46% of cancerous tissues, and 35.98, 0.57, and 3.48% of adjacent non-cancerous tissues, respectively. Firmicutes was statistically significantly more abundant in the gut microbiota of cancerous tissues than that of adjacent non-cancerous tissues (*P* = 0.03), and Proteobacteria was statistically significantly less abundant in the gut microbiota of cancerous tissues than that of adjacent non-cancerous tissues (*P* < 0.01). No statistically significant differences of Fusobacteria were observed in the cancerous tissues compared with non-cancerous tissues (Figure 2C).

At the genus level, our studies also demonstrated that the microbial composition was significantly different and a greater number of genera were present in cancerous tissues compared to adjacent non-cancerous tissues. Genera *Lactococcus*, *Bacteroides*, *Fusobacterium*, *Prevotella*, and *Streptococcus* exhibited more enriched in cancerous tissues than adjacent non-cancerous tissues. However, *Pseudomonas* were statistically significantly enriched in adjacent non-cancerous tissues compared to cancerous tissues (*P* < 0.001, Figure 2D).

### Comparison of gut microbiota between proximal colon cancer and distal colorectal cancer

**Figure 4:**
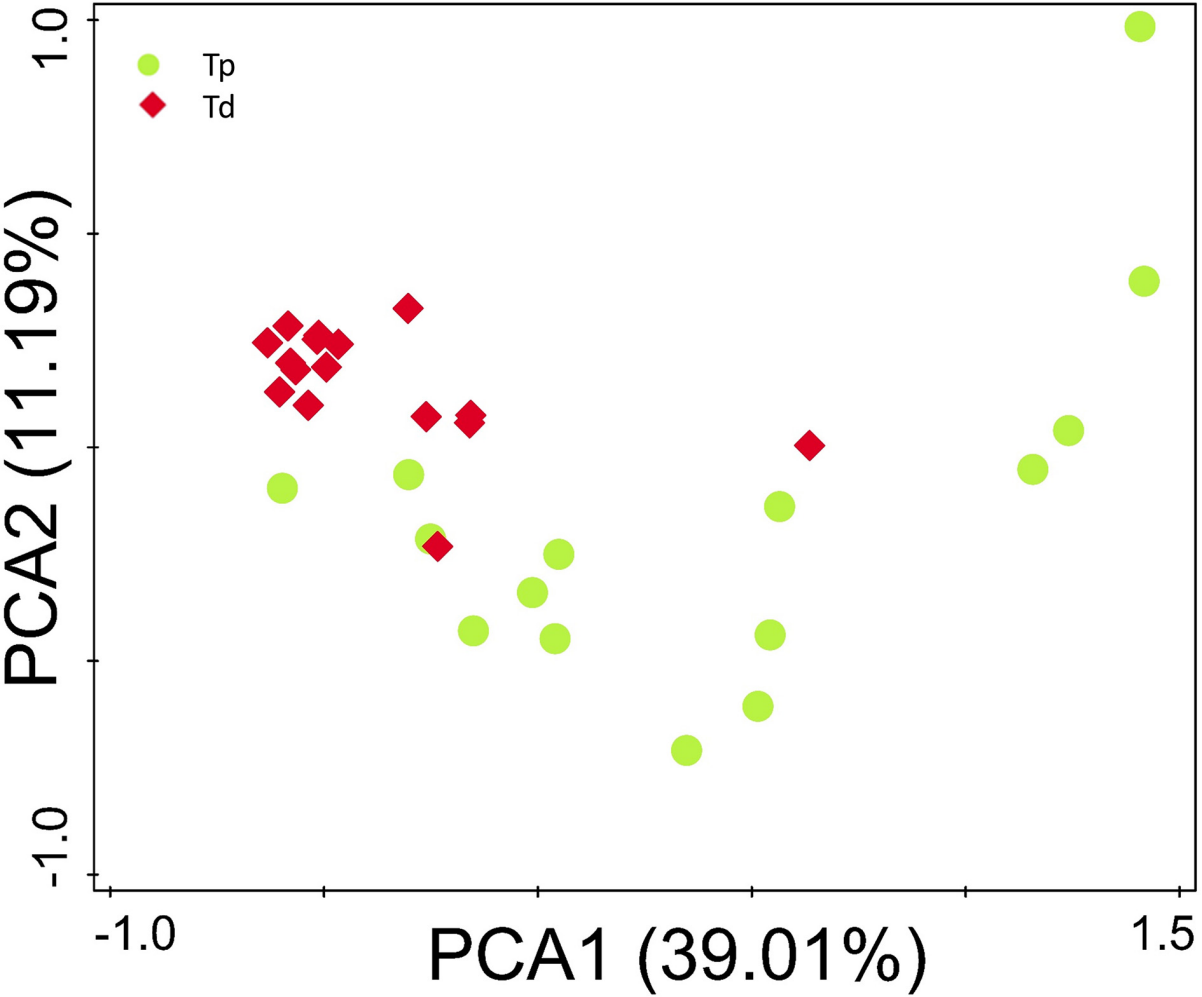
**Principal component analysis (PCA) scores plot based on the relative abundance of OTUs (97% similarity level)**. Each symbol represents a sample. Green circles represent proximal CRC. Red quadrates represent distal CRC.

The microbial composition evaluated in this study resulted as being different from proximal to distal tumors (Figure 2F). Principal component analysis (PCA) based on the relative abundance of genera revealed that a significant separation in bacterial community composition between proximal and distal tumors using the first two principal component scores of PC1 and PC2 (39.07 and 11.2% of explained variance, respectively; Figure 4). We found that the dominant phyla were Firmicutes, Bacteroidetes, and Proteobacteria. They acccounted for above 98% of all phylums.

At genus level, bacterial genera were analyzed that were presented at a relative abundance >0.1%, which accounted for over 97% of total microbiota. Four hundred and sixty-eight bacterial phylotypes were identified from this study. Among of which, 279 (59.6%) presented in proximal colon, 335 (71.6%) in distal colon, which is concordant with the result of diversity index analyses of the gut flora, indicating an increasing microbial richness from proximal colon to rectal cancer. In proximal tumors, *Prevotella, Pyramido- bacterium*, *Selenomonas*, and *Peptostreptoccus* exhibited a relatively higher abundance. While in distal colorectal tumors, *Fusobacterium*, *Escherichia-Shigella*, and *Leptotrichia* were relatively abundant compared with proximal cancerous tissue.

## Discussion

In summary, in this study, we first compared the mucosa-associated microbiota composition between healthy individuals and CRC patients using the platform of Roche 454 sequencer. We also analyzed the mucosa-associated microbial composition in cancerous tissue and the adjacent non-neoplastic tissue. We revealed significant difference of gut microbiota in CRC patients compared with healthy individuals. The relative abundance of dominant phyla Firmicutes, Proteobacteria, Fusobacteria, and dominant genera *Lactococcus*, *Fusobacterium*, *Escherichia-Shigella*, *Peptostrepto- coccus* were all different. A significantly higher abundance of Firmicutes and Fusobacteria in cancerous tissues were found than that in healthy individuals, while Proteobacteria was less abundant in CRC group. Firmicutes, which as part of the gut microbiome has been shown to be involved in energy resorption (Costello et al., 2010), is highly diverse in phenotypic characteristics. Members of the phylum display a disparate distribution in which some species are enriched in the tumor tissue whereas others inhabit healthy gut. For instance, Moore found *Eubacterium rectale*, *Eubacterium eligens* of *Firmicutes* have a significant correlation with CRC (Moore and Holdeman Moore, 1986). In contrast, Proteobacteria, which was less abundant in CRC patients, are generally regarded as gut commensals with potential-pathogenic features (Joly et al., 2010). Our finding indicated that bacteria belonging to the same taxonomic clade can play distinct functional roles in gut environment depending on their functional repertoire, including toxins, virulence factors, and other factors that promote interactions between the bacteria and their microenvironment.

The most prominent and consistent findings is the enrichment of *Fusobacterium* within the tumor microenvironment. It has been reported that *Fusobacterium* may be associated with inflammatory bowel diseases (IBD), including both ulcerative colitis and Crohn's disease (Neut et al., 2002; Ohkusa et al., 2002; Strauss et al., 2011), these two diseases are known risk factors for colorectal cancer. In further study, *Fusobacterium* species can promote host proinflammatory response (Moore and Moore, 1994) and possess virulence characteristics that promote their adhesiveness to host epithelial cells (Bachrach et al., 2005; Uitto et al., 2005) and their ability to invade into epithelial cells (Castellarin et al., 2011). Tomomitsu found *Fusobacterium* enrichment is associated with specific molecular subsets of CRCs including CIMP positivity, TP53 wild type, hMLH1 methylation positivity, MSI and CHD7/8 mutation positivity (Tahara et al., 2014). *Lactococcus*, which are generally regarded as gut commensals with probiotic features, were over-represented in CRC patients, suggesting the microbial shifts are caused by the quite dramatic physiological and metabolic alterations that result from colon carcinogenesis itself (Sansonetti, 2004; Hirayama et al., 2009). According to driver–passenger model for colorectal cancer (Tjalsma et al., 2012), these species may be regarded as CRC bacterial passengers. As expected, our findings indicated that the structure of mucosa-associated microbiota in cancerous tissue differs significantly from that of the adjacent non-neoplastic tissue. The microbial communities of cancerous tissues were more similar to each other than that of tumor and matched non-cancerous colon from a given patient tumors. Proximal colon cancer tissues microbial structures also exhibited similarity with that of distal colon cancer, this is partially due to the unavoidable continuity that the feces pass through gut. Hierarchical clustering analysis of the species-specific relative abundances of microbial sequences confirmed this result.

Harold and his colleagues have proposed a driver–passenger model for colorectal cancer, that is, CRC can be initiated by “driver” bacteria, which are eventually replaced by “passenger” bacteria that either promote or stall tumorigenesis. The bacterial drivers and passengers have distinct temporal roles in CRC pathogenesis. We hypothesized that there are also certain bacteria that are directly pro-oncogenic and capable of remodeling the mucosal immune response and colonic bacterial community to further promote CRC. For example, *Pseudomonas*, which belongs to genus of Gram-negative, aerobic gammaproteobacteria, increasingly recognized as an emerging opportunistic pathogen of clinical relevance (Decker and Palmore, 2014), is significantly less abundant in cancerous tissue compared to normal tissue. This finding was confirmed in mucosa-adherent microbiota of CRC patients compared to adjacent non-cancerous tissues and healthy controls. Other remarkable observation concerned the decreased presence of members of *Escherichia*, *Citrobacter*, *Shigella*, *Flavobacterium*, *Acinetobacter*, *Chryseobacterium* in tumor tissue of the investigated CRC patients. *Shigella*, which were recognized as aetiological agents of human diarrheal disease by a CRC-driving mechanism to prolong inflammatory response, could increase an individual's susceptibility to CRC (Paul et al., 2006; DuPont, 2009; Maggio et al., 2009; Housseau and Sears, 2010). Moreover, it was recently found that Shigella were over-represented in the mucosa-associated microbiome of patients with an adenoma (Maggio et al., 2009; Shen et al., 2010). In addition, certain *Escherichia coli* strains, which harbor the polyketide synthetase (pks) island encoding genotoxin, can induce single-strand DNA breaks (Nougayrède et al., 2006), Subsequent activating DNA damage- induced signaling pathways and increase the mutation rate of infected cells. Therefore, these species may be associated with the early stages of CRC, including adenomas, and then disappear from cancerous tissue as the disease progresses. This data suggest that these potential pathogens are part of the intrinsic bacterial drivers of CRC patients, but outcompeted by commensal bacteria during disease progression. This observation is consistent with our hypothesis.

However, the dramatic physiological and metabolic alterations that occur during colon carcinogenesis may disrupt the structure of indigenous bacterial communities. Some species rarely colonizing colon will be adapted to the new environment. For example, *Fusobacterium* is a genus of obligate anaerobic, Gram-negative bacteria that usually colonize in the oral cavity of nearly all humans, some strains of *Fusobacterium* contribute to the development of dental plaques and periodontal disease (Allen et al., 2011; He et al., 2011). These bacteria are poor colonizers of healthy colon mucosa and cannot breach the intact colon wall. However, when an inflammation, adenoma or carcinoma develops, the deteriorated microenvironment of the colon wall may allow these microorganisms including *Fusobacterium*, *Peptostreptococcus*, and *Lactococcus* to access and adhere the basement membrane. We speculated one of reasons may be the formation of local anaerobic microenvironment induced by aerobic bacteria such as pseudomonas, which is suitable for these potential pathogens to colonize. Different from *Fusobacterium*, however, *Lactococcus*, which produce a single product-lactic acid, plays a probiotic role in colon. It is not yet clear whether these bacterial passengers merely benefit from the CRC microenvironment or they also play an active part in disease progression.

Additionally, our study also demonstrates several significant differences between proximal and distal CRC in the mucosal microbial composition. The relative abundance of dominant genus *Lactococcus*, *Fusobacterium*, *Pseudomonas*, and *Flavobacterium* are similar between proximal and distal colon. Interestingly, a common characteristic of these bacteria is they all are “passenger bacteria” as discussed in the previous section. One interpretation of this observation may be that the similar tumor microenvironment develop during colon carcinogenesis, including pH, temperature and oxygen. In contrast, *Escherichia-Shigella*, which may belong to potential pathogen of CRC, is highly enriched in proximal colon. The highly enriched Gram-negative bacteria *Bacteroides* in distal colon may impart both beneficial and detrimental effects on host physiology through their colitogenic or probiotic potential (Zhu et al., 2014). One of human colonic commensal, *enterotoxigenic B. Fragilis* (ETBF) have been demonstrated to produce a metalloprotease (also known as fragilysin) in colon cancer patients (Toprak et al., 2006; Wu et al., 2006). This pathogen can facilitate tumorigenesis by triggering augmented expression of inter leukin-17 (IL-17) by T helper 17 (T_H_17) cells in the lamina propria in a mouse model of ETBF-induced colitis and carcinogenesis (Wu et al., 2009). In addition, *Prevotella*, which have been reported in the oral and gastric cavities (Dicksved et al., 2009), was highly enriched in proximal colon cancer that appeared to be linked with elevated IL17 producing cells in the mucosa of CRC patients (Sobhani et al., 2011).

In summary, our study suggest that gut dysbiosis are associated with CRC risk largely through metabolic exchange or direct interaction with the host. We speculate two kinds of functionally different bacteria present within tumor microenvironment during the process of tumorigenesis. One is certain potential pro-oncogenic pathogens such as ETBF and *Escherichia-Shigella*, which can promote tumorigenesis as driver bacteria. While an altered tumor microenvironment develops with the disease progression, the other kind of bacteria, which may play either tumor-promoting or tumor-suppressing role, can be more adapted of the new environment than driver bacteria and survive finally. Our findings also reveal species-specific alterations between proximal and distal colon cancer. Still, further studies on wide sample sizes will be needed to identify the role of the different microbiota. Therefore, our results will be useful to promote the development of novel bacteria-related diagnostic tools and therapeutic interventions.

### Conflict of interest statement

The authors declare that the research was conducted in the absence of any commercial or financial relationships that could be construed as a potential conflict of interest.

## Supplementary material

Figure S1 **Rarefare curve of samples**.

## References

[B1] Allen V. E. Strauss J. Chadee K. (2011). *Fusobacterium nucleatum*: an emerging gut pathogen? Gut Microbes 2, 294–298. 10.4161/gmic.2.5.18603 22067936

[B2] Bachrach G. Ianculovici C. Naor R. Weiss E. I. (2005). Fluorescence-based measurements of *Fusobacterium nucleatum* coaggregation and of fusobacterial attachment to mammalian cells. FEMS Microbiol. Lett. 248, 235–240. 10.1016/j.femsle.2005.05.055 15993010

[B3] Borugian M. J. Sheps S. B. Whittemore A. S. Wu A. H. Potter J. D. Gallagher R. P. (2002). Carbohydrates and colorectal cancer risk among Chinese in North America Cancer. Epidemiol. Biomarkers Prev. 11, 187–193. 11867506

[B4] Brenner H. Kloor M. Pox C. P. (2014). Colorectal cancer. Lancet 383, 1490–1502. 10.1016/S0140-6736(13)61649-9 24225001

[B5] Bufill J. A. (1990). Colorectal cancer: evidence for distinct genetic categories based on proximal or distal tumor location. Ann. Intern. Med. 113, 779–788. 10.7326/0003-4819-113-10-779 2240880

[B6] Castellarin M. Warren R. L. Freeman J. D. Dreolini L. Krzywinski M. Strauss J.. (2011). *Fusobacterium nucleatum* infection is prevalent in human colorectal carcinoma. Genome Res. 22, 299–306. 10.1101/gr.126516.111 22009989 PMC3266037

[B7] Cole J. R. Wang Q. Cardenas E. Fish J. Chai B. Farris R. J.. (2009). The Ribosomal Database Project: improved alignments and new tools for rRNA analysis. Nucleic Acids Res. 37, D141–D145. 10.1093/nar/gkn879 19004872 PMC2686447

[B8] Costello E. K. Gordon J. I. Secor S. M. Knight R. (2010). Postprandial remodeling of the gut microbiota in Burmese pythons. ISME J. 4, 1375–1385. 10.1038/ismej.2010.71 20520652 PMC3923499

[B9] Cuevas-Ramos G. Petit C. R. Marcq I. Boury M. Oswald E. Nougayrède J. P. (2010). *Escherichia coli* induces DNA damage *in vivo* and triggers genomic instability in mammalian cells. Proc. Natl. Acad. Sci. U.S.A. 107, 11537–11542. 10.1073/pnas.1001261107 20534522 PMC2895108

[B10] Decker B. K. Palmore T. N. (2014). Hospital water and opportunities for infection prevention. Curr. Infect. Dis. Rep. 16:432. 10.1007/s11908-014-0432-y 25217106 PMC5583638

[B11] DeSantis T. Z. Hugenholtz P. Larsen N. Rojas M. Brodie E. L. Keller K.. (2006). Greengenes, a chimera-checked 16S rRNA gene database and workbench compatible with ARB. Appl. Environ. Microbiol. 72, 5069–5072. 10.1128/AEM.03006-05 16820507 PMC1489311

[B12] Dicksved J. Lindberg M. Rosenquist M. Enroth H. Jansson J. K. Engstrand L. (2009). Molecular characterization of the stomach microbiota in patients with gastric cancer and in controls. J. Med. Microbiol. 58, 509–516. 10.1099/jmm.0.007302-0 19273648

[B13] DuPont H. L. (2009). Bacterial diarrhea. NEJM 361, 1560–1569. 10.1056/NEJMcp0904162 19828533

[B14] Edgar R. C. (2013). UPARSE: highly accurate OTU sequences from microbial amplicon reads. Nat. Methods 10, 996–998. 23955772 10.1038/nmeth.2604

[B15] Fish J. A. Chai B. Wang Q. Sun Y. Brown C. T. Tiedje J. M.. (2013). FunGene: the functional gene pipeline and repository. Front. Microbiol. 4:291. 10.3389/fmicb.2013.00291 24101916 PMC3787254

[B16] Hamady M. Walker J. J. Harris J. K. Gold N. J. Knight R. (2008). Error-correcting barcoded primers for pyrosequencing hundreds of samples in multiplex. Nat. Methods 5, 235–237. 10.1038/nmeth.1184 18264105 PMC3439997

[B17] He X. Hu W. Kaplan C. W. Guo L. Shi W. Lux R. (2011). Adherence to streptococci facilitates *Fusobacterium nucleatum* integration into an oral microbial community. Microb. Ecol. 63, 532–542. 10.1007/s00248-011-9989-2 22202886 PMC3313671

[B18] Hirayama A. Kami K. Sugimoto M. Sugawara M. Toki N. Onozuka H.. (2009). Quantitative metabolome profiling of colon and stomach cancer microenvironment by capillary electrophoresis time-of-flight mass spectrometry. Cancer Res. 69, 4918–4925. 10.1158/0008-5472.CAN-08-4806 19458066

[B19] Housseau F. Sears C. L. (2010). Enterotoxigenic Bacteroides fragilis (ETBF)-mediated colitis in Min (Apc^+/−^) mice: a human commensal-based murine model of colon carcinogenesis. Cell Cycle 9, 3–5. 10.4161/cc.9.1.10352 20009569 PMC3021131

[B20] Huycke M. M. Abrams V. Moore D. R. (2002). *Enterococcus faecalis* produces extracellular superoxide and hydrogen peroxide that damages colonic epithelial cell DNA. Carcinogenesis 23, 529–536. 10.1093/carcin/23.3.529 11895869

[B21] Joly F. Mayeur C. Bruneau A. Noordine M. L. Meylheuc T. Langella P.. (2010). Drastic changes in fecal and mucosa-associated microbiota in adult patients with short bowel syndrome. Biochimie 92, 753–761. 10.1016/j.biochi.2010.02.015 20172013

[B22] Kõljalg U. Nilsson R. H. Abarenkov K. Tedersoo L. Taylor A. F. Bahram M.. (2013). Towards a unified paradigm for sequence−based identification of fungi. Mol. Ecol. 22, 5271–5277. 10.1111/mec.12481 24112409

[B23] Kostic A. D. Gevers D. Pedamallu C. S. Michaud M. Duke F. Earl A. M.. (2011). Genomic analysis identifies association of Fusobacterium with colorectal carcinoma. Genome Res. 22, 292–298. 10.1371/journal.pone.0020447 22009990 PMC3266036

[B24] Maggio P. L. Treuting P. Bielefeldt O. H. Seamons A. Drivdahl R. Zeng W.. (2009). Bacterial infection of Smad3/Rag2 double-null mice with transforming growth factor-βdysregulation as a model for studying inflammation-associated colon cancer. Am. J. Pathol. 174, 317–329. 10.2353/ajpath.2009.080485 19119184 PMC2631344

[B25] Marchesi J. R. Dutilh B. E. Hall N. Peters W. H. M. Roelofs R. Boleij A.. (2011). Towards the human colorectal cancer microbiome. PLoS ONE 6:e20447. 10.1371/journal.pone.0020447 21647227 PMC3101260

[B26] McMichael A. J. Potter J. D. (1985). Diet and colon cancer: integration of the descriptive, analytic, and metabolic epidemiology. Natl. Cancer Inst. Monogr. 69, 223–228. 3834337

[B27] Moore W. E. C. Holdeman Moore L. V. (1986). Genus *Eubacterium* Prevot 1938 294^AL^, in Bergey's Manual of Systematic Bacteriology, Vol. 2 eds Sneath P. H. A. Mair N. S. Sharpe M. E. Holt J. G. (Baltimore, Md: Williams & Wilkins), 1353–1373.

[B28] Moore W. E. Moore L. V. (1994). The bacteria of periodontal diseases. Periodontol. 2000. 5, 66–77. 10.1111/j.1600-0757.1994.tb00019.x 9673163

[B29] Neut C. Bulois P. Desreumaux P. Membre J. M. Lederman E. Gambiez L.. (2002). Changes in the bacterial flora of the neoterminal ileum after ileocolonic resection for Crohn's disease. Am. J. Gastroenterol. 97, 939–946. 10.1111/j.1572-0241.2002.05613.x 12003430

[B30] Nougayrède J. P. Homburg S. Taieb F. Boury M. Brzuszkiewicz E. Gottschalk G.. (2006). *Escherichia coli* induces DNA double-strand breaks in eukaryotic cells. Science 313, 848–851. 10.1126/science.1127059 16902142

[B31] Ohkusa T. Sato N. Ogihara T. Morita K. Ogawa M. Okayasu I. (2002). Fusobacterium variumlocalized in the colonic mucosa of patients with ulcerative colitis stimulates species-specific antibody. J. Gastroenterol. Hepatol. 17, 849–853. 10.1046/j.1440-1746.2002.02834.x 12164960

[B32] Paul R. M. Laurie E. H. Darrell B. O. Whitney S. H. Daniel C. B. Charles O. E.. (2006). Transforming growth factor-β induces development of the TH17 lineage. Nature 441, 231–234. 10.1038/nature04754 16648837

[B33] Qin J. J. Li R. Q. Raes J. Arumugam M. Burgdorf K. S. Manichanh C.. (2010). A human gut microbial gene catalogue established by metagenomic sequencing. Nature 64, 59–65. 20203603 10.1038/nature08821 PMC3779803

[B34] Quast C. Pruesse E. Yilmaz P. Gerken J. Schweer T. Yarza P.. (2013). The SILVA ribosomal RNA gene database project: improved data processing and web-based tools. Nucleic Acids Res. 41, D590–D596. 10.1093/nar/gks1219 23193283 PMC3531112

[B35] Ray K. (2011). Colorectal cancer: *Fusobacterium nucleatum* found in colon cancer tissue-could an infection cause colorectal cancer? Nat. Rev. Gastroenterol. Hepatol. 8, 662. 10.1038/nrgastro.2011.208 22083120

[B36] Sansonetti P. J. (2004). War and peace at mucosal surfaces. Nat. Rev. Immunol. 4, 953–964. 10.1038/nri1499 15573130

[B37] Schwabe R. F. Jobin C. (2013). The microbiome and cancer. Nat. Rev. Cancer 13, 800–812. 10.1038/nrc3610 24132111 PMC3986062

[B38] Shen X. J. Rawls J. F. Randall T. Burcal L. Mpande C. N. Jenkins N.. (2010). Molecular characterization of mucosal adherent bacteria and associations with colorectal adenomas. Gut Microbes 1, 138–147. 10.4161/gmic.1.3.12360 20740058 PMC2927011

[B39] Sobhani I. Tap J. Roudot-Thoraval F. Roperch J. P. Letulle S. Langella P.. (2011). Microbial dysbiosis in colorectal cancer (CRC) patients. PLoS ONE 6:e16393. 10.1371/journal.pone.0016393 21297998 PMC3029306

[B40] Srikanth C. V. McCormick B. A. (2008). Interactions of the intestinal epithelium with the pathogen, and the indigenous microbiota: a three-way crosstalk. Interdiscip. Perspect. Infect. Dis. 2008, 626827. 10.1155/2008/626827 19259328 PMC2648619

[B41] Strauss J. Kaplan G. G. Beck P. L. Rioux K. Panaccione R. Devinney R.. (2011). Invasive potential of gut mucosa-derived *Fusobacterium nucleatum* positively correlates with IBD status of the host. Inflamm. Bowel Dis. 17, 1971–1978. 10.1002/ibd.21606 21830275

[B42] Tahara T. Yamamoto E. Suzuki H. Maruyama R. Chung W. Garriga J.. (2014). Fusobacterium in colonic flora and molecular features of colorectal carcinoma. Cancer Res. 74, 1311–1318. 10.1158/0008-5472.CAN-13-1865 24385213 PMC4396185

[B43] Takada H. Ohsawa T. Iwamoto S. Yoshida R. Nakano M. Imada S.. (2002). Changing site discription of colorectal cancer in Japan. Dis. Colon Rectum 45, 1249–1254. 10.1007/s10350-004-6400-0 12352244

[B44] Tjalsma H. Boleij A. Marchesi J. R. Dutilh B. E. (2012). A bacterial driver-passenger model for colorectal cancer: beyond the usual suspects. Nat. Rev. Microbiol. 10, 575–582. 10.1038/nrmicro2819 22728587

[B45] Toprak N. U. Yagci A. Gulluoglu B. M. Akin M. L. Demirkalem P. Celenk T.. (2006). A possible role of Bacteroides fragilisenterotoxin in the aetiology of colorectal cancer. Clin. Microbiol. Infect. 12, 782–786. 10.1111/j.1469-0691.2006.01494.x 16842574

[B46] Uitto V. J. Baillie D. Wu Q. Gendron R. Grenier D. Putnins E. E.. (2005). *Fusobacterium nucleatum* increases collagenase 3 production and migration of epithelial cells. Infect. Immun. 73, 1171–1179. 10.1128/IAI.73.2.1171-1179.2005 15664960 PMC547012

[B47] Uronis J. M. Mühlbauer M. Herfarth H. H. Rubinas T. C. Jones G. S. Jobin C. (2009). Modulation of the intestinal microbiota alters colitis-associated colorectal cancer susceptibility. PLoS ONE 4:e6026. 10.1371/journal.pone.0006026 19551144 PMC2696084

[B48] Wang X. Allen T. D. May R. J. Lightfoot S. Houchen C. W. Huycke M. M. (2008). *Enterococcus faecalis* induces aneuploidy and tetraploidy in colonic epithelial cells through a bystander effect. Cancer Res. 68, 9909–9917. 10.1158/0008-5472.CAN-08-1551 19047172 PMC2596646

[B49] Wang X. M. Huycke M. M. (2007). Extracellular superoxide production by *Enterococcus faecalis* promotes chromosomal instability in mammalian cells. Gastroenterology 132, 551–561. 10.1053/j.gastro.2006.11.040 17258726

[B50] Wei H. Dong L. Wang T. Zhang M. Hua W. Zhang C.. (2010). Structural shifts of gut microbiota as surrogate endpoints for monitoring host health changes induced by carcinogen exposure. FEMS Microbiol. Ecol. 73, 577–586. 10.1111/j.1574-6941.2010.00924.x 20629751

[B51] West D. W. Slattery M. L. Robison L. M. Schuman K. L. Ford M. H. Mahoney A. W.. (1989). Dietary intake and colon cancer: sex- and anatomic site-specific associations. Am. J. Epidemiol. 130, 883–894. 2554725 10.1093/oxfordjournals.aje.a115421

[B52] Wu G. D. Chen J. Hoffmann C. Bittinger, K, Chen Y. Y. Keilbaugh S. A.. (2011). Linking long-term dietary patterns with gut microbial enterotypes. Science 334, 105–108. 10.1126/science.1208344 21885731 PMC3368382

[B53] Wu S. Rhee K. J. Albesiano E. Rabizadeh S. Wu X. Yen H. R.. (2009). A human colonic commensal promotes colon tumorigenesis via activation of T helper type 17 T cell responses. Nat. Med. 15, 1016–1022. 10.1038/nm.2015 19701202 PMC3034219

[B54] Wu S. Shin J. Zhang G. Cohen M. Franco A. Sears C. L. (2006). The *Bacteroides fragilis* toxin binds to a specific intestinal epithelial cell receptor. Infect. Immun. 74, 5382–5390. 10.1128/IAI.00060-06 16926433 PMC1594844

[B55] Zhu Q. Jin Z. Wu W. Gao R. Guo B. Gao Z.. (2014). Analysis of the intestinal lumen microbiota in an animal model of colorectal cancer. PLoS ONE. 9:e90849. 10.1371/journal.pone.0090849 24603888 PMC3946251

