# Supplementary figures and images for: Microbiota disbiosis is associated with colorectal cancer
Source: Front Microbiol. 2015 Feb 2;6:20. doi: 10.3389/fmicb.2015.00020 (PMC4313696; doi:10.3389/fmicb.2015.00020)

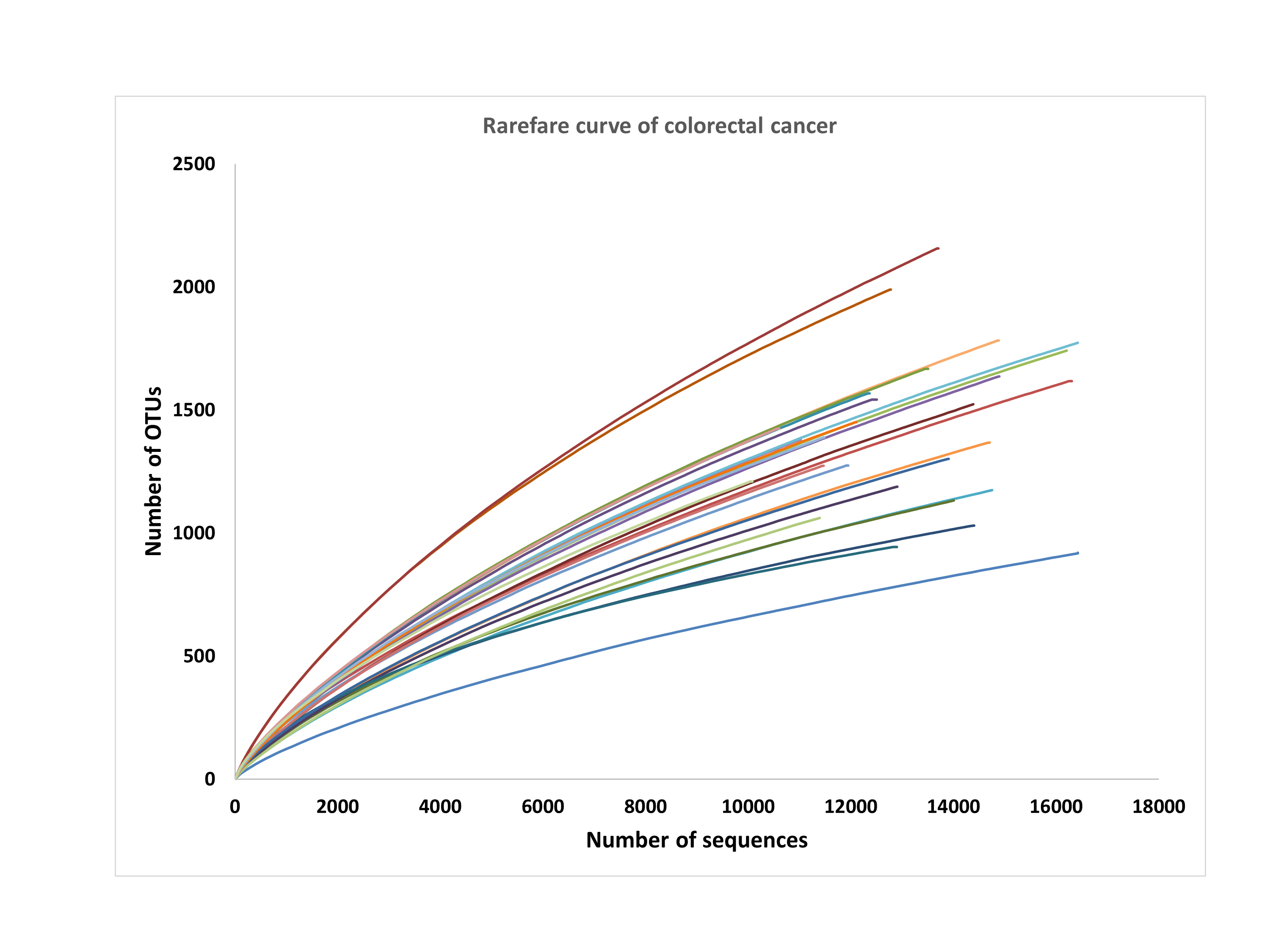

Supplement: Figure S1 — Rarefare curve of samples. [file Image1.TIF]
